# Asthma in Competitive Cross-Country Skiers: A Systematic Review and Meta-analysis

**DOI:** 10.1007/s40279-020-01334-4

**Published:** 2020-09-11

**Authors:** Rikhard Mäki-Heikkilä, Jussi Karjalainen, Jari Parkkari, Maarit Valtonen, Lauri Lehtimäki

**Affiliations:** 1 Faculty of Medicine and Health Technology, Tampere University, Tampere, Finland; 2 Allergy Centre, Tampere University Hospital, Tampere, Finland; 3 Tampere Research Center of Sports Medicine, UKK Institute, Tampere, Finland; 4 KIHU, Research Institute for Olympic Sports, Jyväskylä, Finland

## Abstract

**Introduction:**

In cross-country skiing, the repetitive ventilation of large amounts of cold and dry air strains the airways. The aim of this systematic review was to establish an overview of the current literature on asthma in cross-country skiers, biathletes and ski-orienteers.

**Methods:**

Six databases were searched on August 29, 2019. The search yielded 2161 articles. Thirty articles fulfilled the search criteria and were pooled together for a qualitative synthesis. Eight articles were included in the meta-analysis on the prevalence of asthma and the use of asthma medication.

**Results:**

According to the meta-analysis, the prevalence of self-reported physician-diagnosed asthma in skiers was 21% (95% CI 14–28%). The onset age of asthma was higher in skiers than in non-skiers with asthma. The prevalence of asthma medication use was on average 23% (CI 95% 19–26%). Several studies reported that asthma was underdiagnosed in skiers, as previously healthy skiers without a prior asthma diagnosis or medication use were frequently found to fulfill diagnostic criteria for asthma according to lung function tests. Studies using bronchial biopsy demonstrated that eosinophilic asthma is not detected in skiers with asthma as often as it is in non-skiers with asthma and that there are signs of airway inflammation even in non-asthmatic skiers.

**Conclusion:**

Our findings suggest that the accuracy and coverage of diagnosing asthma in skiers has improved over the recent decades. However, the optimal treatment and natural course of asthma in this population remain unclear. Future research should investigate how the intensity of training, airway infections and their treatment affect the development of asthma among skiers.

**PRD registration number:**

CRD42017070940.

**Electronic supplementary material:**

The online version of this article (10.1007/s40279-020-01334-4) contains supplementary material, which is available to authorized users.

## Key Points

**Table:** 

The prevalence of asthma in cross-country skiers is 21 %, which is higher than that in the general population.
There is an indication that asthma is underdiagnosed among skiers, especially during the previous decades, as many skiers without previous diagnosis of asthma or asthma medication fulfilled criteria for asthma according to lung function tests.
There is a need for international consensus over the criteria of asthma and its treatment in athletes to avoid both over and under diagnosis of asthma.
The usual onset age of asthma in cross-country skiers is 10–17 years of age, which is different from that in the general population, for whom the onset more often occurs in early childhood.
The prevalence of asthma and use of asthma medication (21 % vs. 23 %) were similar, suggesting that there is no remarkable overuse of asthma medication among skiers. However, the data is limited on the use of asthma medication in skiers without diagnosis of asthma.
Asthma in skiers seems to be less often eosinophilic and more often neutrophilic compared to asthma in non-skiers.
Due to the high prevalence of asthma in cross-country skiers, regular screening of asthma-like symptoms and lung function could be beneficial to competitive skiers.

## Introduction

Asthma is a heterogeneous disease characterized by variable airway obstruction and is usually associated with chronic airway inflammation. It is defined by a history of respiratory symptoms, such as wheezing, shortness of breath, chest tightness and coughing that varies over time and in intensity, together with variable expiratory airflow limitations [1]. Airway inflammation, airway hyperresponsiveness and bronchoconstriction during or after exercise are common pathophysiological features related to asthma. The diagnosis of asthma is recommended to be based on typical symptoms and objective evidence of variable airway obstruction [1]. The most frequently used methods of diagnosis are spirometry with the bronchodilation test or tests of bronchial hyperresponsiveness (e.g., exercise tests or methacholine challenge tests) [2, 3].

The prevalence of physician-diagnosed asthma is reported to be 4.3% globally [4] and approximately 8–12% in countries, where cross-country skiing is popular, such as Nordic countries, France, and North America [4–7]. The prevalence of asthma among athletes varies notably between sports. Athletes who engage in sports with high ventilatory requirements, such as endurance sports, have a higher prevalence of asthma than athletes who engage in low ventilatory sports, such as archery or shooting [8]. Additionally, the use of asthma medications, such as β_2_-agonists, varies between sports. In the Winter Olympic Games from 2002 until 2010, the approved usage rate for β2-agonists was the highest in cross-country skiing and Nordic combined (17.2% and 12.9%, respectively), while the usage rate was the lowest in ski jumping and the luge (3.1% and 2.7%, respectively) [9].

Cross-country skiing is a demanding Olympic winter sport. High maximal oxygen uptake and anaerobic capacity are needed in addition to high levels of upper body power [10]. Minute ventilation in elite skiers may well exceed 200 l/min, and their forced vital capacity (FVC) and forced expiratory volume in one second (FEV1) often exceed normal values [11]. Skiers may train in very dry and cold air that reaches extremely low temperatures. The International Ski Federation (FIS) set a temperature limit for organizing competitions, and it is currently − 20 °C (− 4 °F) [12]. The absolute humidity of cold air is very low, which may exacerbate symptoms and promote bronchial constriction in people with pre-existing asthma [13]. Repeated exposure to cold air over many years of intensive training may also cause airway inflammation and asthma [14, 15].

To the best of our knowledge, no systematic reviews on asthma in cross-country skiers have been published. Our aim was to establish a systematic review of the literature on all available aspects of asthma in cross-country skiers.

## Methods

This systematic review followed the Preferred Reporting Items for Systematic Reviews and Meta-Analysis (PRISMA) guidelines [16] and was registered in the PROSPERO database (registration number CRD42017070940).

### Literature search

Original articles from PubMed, EBSCO Academic Search Premier, Web of Science, Scopus, Cochrane Library and clinicaltrials.gov were searched. The search date was August 29, 2019. No language or time filters were used. The details of the search strategy are presented in Supplementary File 1. After study selection, the references from these articles were screened for further references.

### Study inclusion

Three authors (RM-H, JK, LL) independently screened the titles and abstracts of the studies. The studies had to meet the following criteria to be included: (1) The study participants were active, competitive cross-country skiers, biathletes, Nordic combined athletes or ski-orienteers, i.e., athletes who compete using cross-country skis. (2) The study produced new original data on the prevalence, incidence or pathophysiology of asthma, physiological phenomena related to asthma or asthma-related symptoms or asthma medication.

The exclusion criteria for our review were as follows: (1) the study population included skiers, but their results were not reported separately from those of other athletes, and (2) the subjects were recreational skiers not competitive skiers.

### Data extraction

From each included study, we extracted the first author, country of the study, publication year, title, study population and characteristics, test protocols, prevalence of self-reported or physician-diagnosed asthma, asthma-related symptoms and environmental factors, allergies and other respiratory diseases, study funding and conflicts of interest. One reviewer (RM-H) extracted the data, and two other authors (JK and LL) verified the correctness of the collected data.

### Quality appraisal and data analyses

Three authors (RM-H, JK, LL) reviewed the risk of bias in the included studies in collaboration using the Cochrane risk of bias tool. Meta-analyses were conducted by calculating Freeman–Tukey-transformed [62] proportions and summary estimates with a random effects model using a restricted maximum likelihood (REML)-approach (transformed prevalence of self-reported physician-diagnosed asthma, asthma in skiers with either a self-reported physician diagnosis or a diagnosis based on lung function measurements, and the use of asthma medication). Inverse variance weighting was used to calculate the weights for individual studies. After performing the meta-analyses, individual proportions and summary estimates were back-transformed according to Miller [63]. Forest plots were then drawn using the back-transformed data. The analyses were conducted using R version 3.4.3 [17] and R-package Metafor [18]. Other outcomes are presented narratively.

## Results

**Fig. 1:**
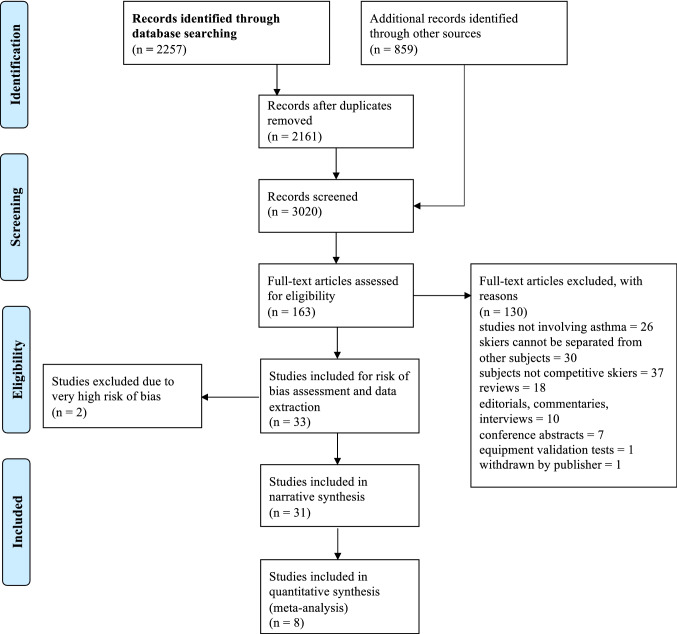
Flowchart of the search performed according to the PRISMA guidelines

The initial search yielded 2257 articles. After the duplicates were removed, the titles and abstracts of the remaining 2161 articles were screened. Based on the screening results, the full texts of 163 articles were retrieved for analysis. On the basis of the full-text analysis, 130 articles were excluded, while 33 articles fulfilled the inclusion criteria. See Supplemental file 3 for reasons. After the risk of bias assessment, 2 articles were excluded due to a very high risk of bias [65, 66] and after careful consideration these studies were excluded from the analyses. Data from 31 articles were extracted for the qualitative synthesis. Eight articles were included in the meta-analysis to assess the prevalence of asthma and the use of asthma medication (Fig. 1) [19–24, 26, 27] After study inclusion, the references from selected articles were screened but no additional records were found.

The publication years of the included studies ranged from 1993 to 2018. Ten articles were epidemiological studies and assessed the prevalence of asthma, onset of asthma, prevalence of asthma-related symptoms and use of asthma medication [19–27, 46]. Fourteen studies assessed different diagnostic tests for asthma [14, 15, 28–39]. Three studies investigated seasonal variability of asthmatic features [40–42], and two studies investigated the effects of bronchodilating asthma medication in healthy skiers [43, 44]. One study investigated the predictive values of asthma-related symptoms [56] and one case study followed four skiers during a 10-year period [45]. The risk of bias assessment results are shown in electronic Supplementary File 2.

### Prevalence of asthma-related symptoms

Asthma-related symptoms include chest tightness, shortness of breath, coughing and wheezing. The prevalence of these symptoms has been studied in different studies using structured questionnaires. In a study by Heir and Oseid [21], 84% of 153 skiers had at least one symptom. Sue-Chu et al. [22] found that 46% of the skiers studied in Norway and 51% of those studied in Sweden had wheezing and breathlessness or chest tightness. In a screening study by Turmel and others, 50% of 44 skiers and biathletes had exercise-induced symptoms [25]. Norqvist et al. [26] found that 22% of the skiers (*n* = 238) had asthma-related symptoms. In the study by Rundell and others [46], 62% of the population of cross-country skiers (*n* = 21) reported at least one symptom.

There was a significant difference in the prevalence of asthma symptoms between sexes among 15–19-year-old skiers (10% in males vs. 30% in females, *p* = 0.001) but not in the 20–34-year-old group (24% in males vs. 29% in females, *p* = 0.582) [26]. In a study by Eklund and colleagues [27], 16% of high-school aged skiers had asthma-related symptoms. They also found a significant difference in the prevalence of symptoms between sexes (9% in males vs. 23% in females, *p* = 0.005) [27].

### Prevalence of asthma

Studies investigating the prevalence of asthma were divided into three categories based on how asthma was defined and diagnosed: (1) self-reported physician-diagnosed asthma; (2) asthma diagnosed based on lung function measures as part of the study; (3) either self-reported physician-diagnosed asthma or asthma diagnosed based on lung function tests in the study.

#### Studies reporting prevalence of self-reported physician-diagnosed asthma

**Fig. 2:**
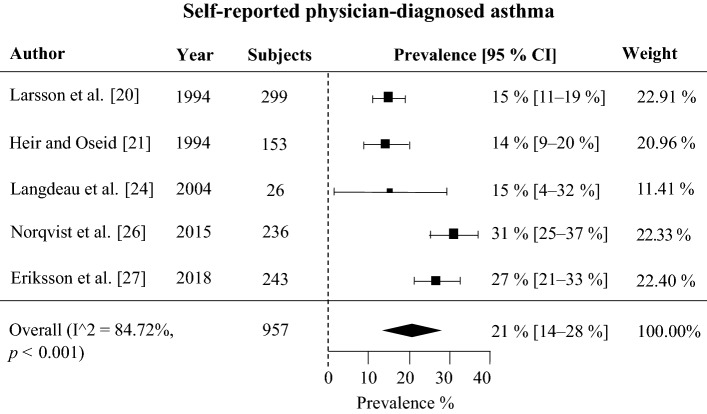
Forest plot of the studies reporting the prevalence of self-reported physician-diagnosed asthma in 957 subjects

Postal self-administered questionnaires were used in five studies to assess the prevalence of self-reported physician-diagnosed asthma (Fig. 2, Table 1). The mean prevalence of asthma in these five studies was 21% (CI 95% 14–28%). Based on these studies, the prevalence of self-reported asthma seems to have increased over time.

**Table 1:** Characteristics of the studies assessing the prevalence of self-reported physician-diagnosed asthma

Year	Author and country	Participants	Sex and age	Diagnostic criteria	Self-reported physician-diagnosed asthma	Risk of bias
1994	Larsson et al. Sweden [20]	299 cross-country skiers from upper secondary schools, national ski teams and the Swedish army; 127 controls from same upper secondary schools	172 M, 127 F, age 18.5 ± 2.4 years (mean)	Yes to question "Do you have asthma diagnosed by a physician?"	15% in skiers, 6% in controls	Low
1994	Heir and Oseid, Norway [21]	153 elite cross-country skiers, 241 controls matched for age, sex and home municipality	106 M, 47 F, age 25.5 years (mean)	Subject reporting asthma diagnosed by physician	14.4% in skiers, 5.0% in controls (*p* = < 0.01)	Low
2004	Langdeau et al. Canada [24]	20 cross-country skiers and 6 biathletes in Quebec	Cannot be extracted	Subject reporting self-reported asthma and/or physician-diagnosed asthma	15.3% (cross-country skiing and biathlon combined)	Moderate. Small sample size
2015	Norqvist et al. Sweden [26]	236 cross-country skiers or biathletes in upper secondary schools, junior and senior national ski teams or universities	Upper secondary school 17 (15–19), national teams 24 (18–34), university athletes (23 (19–31), mean and (range)	Yes to both of the following questions: "Have you ever had asthma?" "Was it diagnosed by a doctor?"	30.9%	Low
2018	Eklund et al. Sweden [27]	244 cross-country skiers, biathletes and ski-orienteers in upper secondary schools	127 M, 117 F, age 16.8 ± 1.2 y	Yes to both of the following questions: "Have you ever had asthma?" "Was it diagnosed by a doctor?"	Total 27%, males 20%, females 34%, controls 19%	Low

Subgroup analysis results for the prevalence of self-reported physician-diagnosed asthma in different age groups and sexes were reported by Norqvist et al. [26]. In skiers between 15 and 19 years of age, the overall prevalence of asthma was 29%, and it was significantly higher in females (38%) than in males (21%, *p* = 0.016). In skiers from 20 to 34 years of age, the prevalence of asthma was 35%, and there was no difference between sexes (32% in males vs. 39% in females, *p* = 0.492).

#### Studies reporting prevalence of asthma based on current lung function measures

We found only one study, where the criteria for asthma were having asthma-like symptoms and current lung function tests as part of the study protocol showed results compatible with asthma. Turmel et al. [25] screened athletes in Quebec, Canada, including cross-country skiers and biathletes, as part of a larger study. They reported a prevalence of 20% of physician-diagnosed asthma based on lung function measures in 44 skiers (Table 2).

**Table 2:** Characteristics of studies assessing the prevalence of asthma based on current lung function measures

Year	Author and country	Participants	Sex and age	Diagnostic criteria	Physician-diagnosed asthma	Risk of bias
2012	Turmel et al. Canada [25]	34 cross-country skiers and 10 biathletes	29 M, 15 F	≥ 12% FEV1 improvement after β_2_-agonist and/or the presence of airway hyperresponsiveness to EVH or methacholine challenge (≤ 4 mg/ml or ≤ 16 mg/ml with active inhaled corticosteroid treatment) and asthmatic symptoms	20%	Moderate. Possible selection bias. Although reversibility was included in the criteria of asthma, only AHR was tested for

**Table 3:** Characteristics of studies assessing prevalence of asthma based on combined criteria of previous physician-diagnosed asthma or current lung function measures

Year	Author and country	Participants	Sex and age	Diagnostic criteria	Total asthma prevalence	Risk of bias
1993	Larsson et al. Sweden [19]	42 cross-country skiers in Stockholm and Östersund	36 M, 6 F, age 24 years (mean)	BHR to methacholine and two asthma-like symptoms OR a previous diagnosis of asthma with active asthma medication use	55%	Moderate. The subjects may not represent the whole skier population well due to the recruitment methods. Methacholine challenge test not identical in different locations
1996	Sue-Chu et al. Norway and Sweden [22]	118 cross-country skiers in senior secondary school in Norway, 38 cross-country skiers in senior secondary school and 15 skiers serving as conscripts in Sweden	Norway 90 M 28 F, age 17.0 ± 1.1 years, Sweden 36 M 17 F, age 18.4 ± 1.4 years	Total cases of asthma defined as current asthma cases or physician-diagnosed asthma cases currently treated with steroids	Norway: 12% Sweden: 42%	Low
2002	Michalak et al. France [23]	180 cross-country skiers or biathletes	121 M 59 F, age 18 ± 2 years (mean)	Increase in FEV_1_ by ≥ 12% or 200 ml in the bronchodilation test or self-reported physician-diagnosed asthma	14%	Low
2012	Turmel et al. Canada [25]	34 cross-country skiers and 10 biathletes	29 M, 15 F	≥ 12% FEV1 improvement after β_2_-agonist and/or the presence of airway hyperresponsiveness to EVH or methacholine challenge (≤ 4 mg/ml or ≤ 16 mg/ml with active inhaled corticosteroid treatment) and asthmatic symptoms	30%	Moderate. Possible selection bias. Although airway reversibility was included in the criteria of asthma, only AHR was tested for

#### Studies reporting prevalence of asthma based on combined criteria of previous physician-diagnosed asthma or current lung function measures

**Fig. 3:**
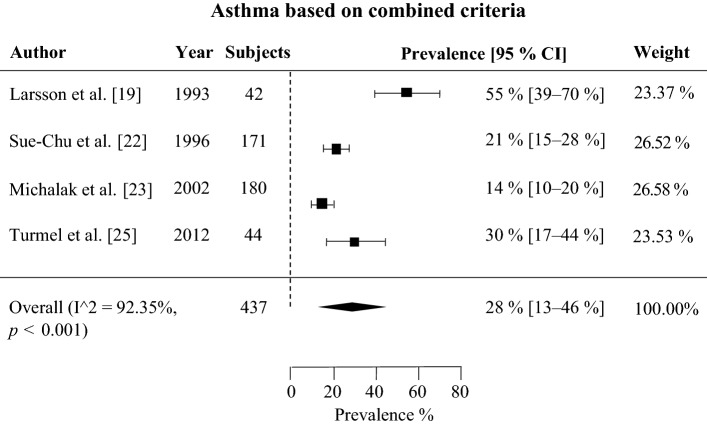
Total asthma prevalence from three studies is 28% in 437 subjects

In three studies, the criteria for asthma were a combination of either having a previous physician-diagnosed asthma or having asthma-like findings in current lung function test [19, 22, 23, 25] (Table 3). The mean prevalence of asthma in these four studies was 28% (CI 95% 13–46%) and is presented in Fig. 3.

### Possible underdiagnosis of asthma among skiers

Several studies have revealed the possibility of asthma being underdiagnosed in skiers. Previously healthy skiers without a prior asthma diagnosis or medication use were found to fulfill diagnostic criteria of asthma according to lung function tests. There seems to be a decreasing trend in the prevalence of undiagnosed asthma in previously healthy skiers, as it decreased from 55 to 20% from 1993–2010, excluding the data from the most recent and relatively small study of 13 subjects [19, 29, 32, 33, 35, 36].

Larsson et al. [19] conducted a study in 1993 in 42 cross-country skiers, where asthma was diagnosed based on positive methacholine challenge test results and the presence of at least two asthma symptoms. Thirty-four percent of the previously healthy skiers fulfilled the diagnostic criteria used. In a later study conducted by Sue-Chu et al. in 1999 [28], 40% (12/30) of previously healthy skiers were considered to have ski asthma. In this study, the diagnosis was also defined as positive methacholine challenge test results and the presence of asthma-like symptoms.

A field exercise challenge was used as the diagnostic test by Ogston and Butcher [32]. They found that 31% (20/91) of the unmedicated high school skiers had exercise-induced bronchoconstriction [32]. A Finnish study by Pohjantähti and others [33] reported that 35% (7/20) of the previously healthy skiers had a decrease in FEV1 by ≥ 10%, a decrease in MMEF (maximal mid-expiratory flow) by ≥ 20% or a decrease in both (2 FEV1, 7 MMEF) with a similar challenge.

In 2010, Sue-Chu et al. [35] compared different diagnostic tests and observed that a total of 25% (12/48) of the previously healthy skiers had bronchial hyperresponsiveness in either the methacholine challenge test or EVH (eucapnic voluntary hyperpnoea) test and fulfilled the criteria for therapeutic use exemption (TUE) at that time.

A British multisport study conducted by Dickinson et al. [36] showed that 62% (8/13) of the biathletes included were previously healthy but had bronchial hyperresponsiveness in the EVH test.

### Risk factors and onset age of asthma or asthma-related symptoms

There is only one study assessing possible risk factors of developing asthma among competitive skiers. Eklund and colleagues reported among 244 skiers from a high school population that family history of asthma and nasal allergy were significant risk factors for asthma both among competitive skiers and non-skiers [27]. It seemed that allergy was not as significant risk factor among skiers as it is among non-skiers.

The age of asthma onset in skiers has been studied in Norway and Sweden in four different studies [19, 21, 26, 27]. The onset age varied from early adolescence to early adulthood, and the onset occurred at a later age in the skiers than in the controls.

Eklund et al. [27] compared junior elite cross-country skiers, biathletes and ski-orienteers to controls in upper secondary schools in Sweden. The median age at onset of asthma in the skier group was significantly higher than that in the controls (12 vs. 8 years, *p* < 0.001). the onset age of asthma was distributed evenly from birth into adolescence in the control group and was concentrated to the 10–15-year-old age range in the skier group. The mean age of the skiers was 16.8 ± 1.2 years.

In another Swedish study conducted by Norqvist et al. [26], the onset age of asthma in both skiers in the 15–19-year-old group and those in the 20–34-year-old group was mostly in early adolescence.

In a study by Heir and Oseid [21], 16 of 22 skiers with self-reported symptoms recalled their onset age of asthma. Fifteen skiers reported an onset age in late adolescence or early adulthood. Only one skier reported the onset of asthma in early childhood. Moreover, Larsson et al. [19] reported that none of the 42 skiers with asthma in their study recalled their onset age of asthma to be during childhood.

### Use of asthma medication among skiers

**Fig. 4:**
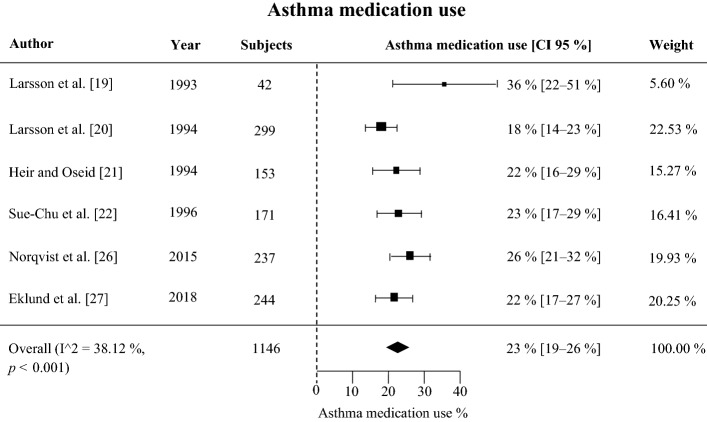
Forest plot of asthma medication use in six studies with 1146 subjects

Six studies reported the use of asthma medication among skiers. Asthma medication use was defined as the use of one or more of the following medications: inhaled bronchodilators (β_2_-agonists or anticholinergic agents), inhaled anti-inflammatories (corticosteroids, cromoglycates), oral theophylline or corticosteroids (Fig. 4, Table 4). The prevalence of asthma medication use in skiers was on average 23% (CI 95% 19–26%) across six studies with 1146 subjects.

**Table 4:** Characteristics of studies assessing the prevalence of asthma medication use among skiers

Year	Author and country	Participants	Sex and age	Use of asthma medication in skiers	Use of asthma medication in controls	Risk of bias
1993	Larsson et al. Sweden [19]	42 skiers and 29 controls	36 M, 6 F, age 24 years (16–50 years)	36% 15/42	0%, healthy controls recruited	Moderate. Small sample size
1994	Larsson et al. Sweden [20]	299 cross-country skiers from upper secondary school, national ski team and Swedish army. 127 controls from same upper secondary schools	172 M, 127 F, age 18.5 ± 2.4 years (mean)	18%	7%	Low
1994	Heir and Oseid, Sweden [21]	153 elite cross-country skiers, 241 controls matched for age, sex and home municipality	106 M, 47 F, age 25.5 years (mean)	22.2% (34/153, 25 regularly, 9 occasionally)	4,6% (11/241, 7 regularly, 4 occasionally)	Low
1996	Sue-Chu et al. Norway and Sweden [22]	118 cross-country skiers in senior secondary school in Norway, 38 skiers in senior secondary school and 15 military conscripts in Sweden	Norway 90 M 28 F, age 17.0 ± 1,1 years, Sweden 36 M 17 F, age 18.4 ± 1,4 years	Total 23% (Sweden 38%, Norway 16%); β_2_-agonist 21% (38 SWE, 14% NOR), ICS (inhaled corticosteroids) 10% (SWE 23%, NOR 4%)	No controls	Low
2015	Norqvist et al. Sweden [26]	237 cross-country skiers or biathletes in upper secondary schools, junior and senior national ski teams or universities	Upper secondary school 17 years (15–19), national teams 24 years (18–34), university athletes (23 (19–31), mean and (range)	15–19 years 25%, 16% M, 35% F (*p* = 0.005); 20–34 years 28%, 18% M, 38% F (*p* = 0.061); total 26%	No controls	Low
2018	Eklund et al. Sweden [27]	244 cross-country skiers, biathletes and ski-orienteers in upper secondary schools	127 males, 117 females, age 16.8 ± 1.2 years	22% (14% M, 23% F) (last 12 mo) (*p* 0.003)	Total 11% (*p* = 0.03 compared to skiers), 8% M, 14% F	Low

### Asthma-related pathophysiological features in skiers

#### Airway inflammation

Airway inflammation in skiers with or without asthma has been investigated using bronchial biopsies in three studies [14, 15, 28], using induced sputum in one study [28] and using exhaled nitric oxide in three studies [29, 37, 39].

The results of the six studies assessing airway inflammation in skiers are summarized in Table 5. In short, studies using bronchial biopsies have demonstrated increased numbers of lymphoid aggregates and inflammatory cells in bronchial mucosa or bronchoalveolar lavage fluid in skiers, most of whom can be considered asthmatic skiers, compared to healthy controls, but these findings were not as marked as those occurring in non-skiing asthmatic controls. However, the exhaled nitric oxide and inflammatory markers in induced sputum were not different between the asthmatic skiers and healthy controls.

**Table 5:** List of studies investigating airway inflammation in cross-country skiers

Year	Author	Subjects	Protocol	Main finding	Risk of bias
1998	Sue-Chu et al. [14]	44 skiers and 12 healthy controls. 59% of the skiers had asthma-like symptoms and were hyperresponsive to methacholine	Bronchial biopsy from second and third generation carinae	Lymphoid aggregates in skiers 64% vs. 25% in controls	Low
1999	Sue-Chu et al. [28]	30 skiers and 10 healthy controls. 63% of the skiers were hyperresponsive to methacholine, and 40% were hyperresponsive and had asthma-related symptoms	Bronchial biopsy and bronchoalveolar lavage	Macroscopic inflammatory index based on the visual evaluation of bronchial mucosa was significantly higher in skiers than in controls (3.1 vs. 1.3, *p* = 0.008). Subjects with “ski asthma” had higher percentages of lymphocytes and lower percentages of macrophages in BAL fluid compared with healthy controls, but these results were not significantly different from those of healthy skiers	Low
1999	Sue-Chu et al. study 2 [29]	44 skiers, 29 mild asthmatic controls and 82 healthy controls. Nine skiers were hyperresponsive to methacholine and had asthmatic symptoms	Exhaled nitric oxide concentration at rest. Expiratory flow rate was set to 250 ml per second	Exhaled nitric oxide concentrations were not different compared to healthy controls (6.5 vs. 5.2 ppb), but asthmatic controls had threefold higher levels compared to skiers (6.5 vs. 19.2 ppb, *p* < 0.01). The atopic skiers had twofold greater exhaled nitric oxide concentrations compared to non-atopic skiers (values not available)	Low
2000	Karjalainen et al. [15]	40 skiers with no prior diagnosis of asthma, 12 asthmatic controls and 12 healthy controls. 75% of the skiers were hyperresponsive to methacholine and 53% were hyperresponsive and had asthma-related symptoms	Bronchial biopsy from second and third generation carinae	Skiers had higher counts of T-lymphocytes, macrophages and eosinophils compared with controls. the counts of macrophage, mast cell and eosinophil cell counts were significantly lower in skiers compared to asthmatic subjects, but neutrophil count was significantly higher in skiers compared to asthmatic controls. Tenascin thickness in subepithelial basement membrane was significantly thicker in skiers compared to healthy controls but in nonhyperresponsive skiers the tenascin thickness was lower compared to patients with asthma	Low
2014	Zebrowska et al. [37]	12 elite female cross-country skiers	Exhaled nitric oxide concentration at rest	Exhaled nitric oxide concentrations were reported to be within a normal range (18.7 ± 4.8 ppb)	High. Selective reporting and measurement times not reported. Possible asthma medication use not reported
2018	Stang et al. [39]	10 skiers and 10 swimmers with previous asthma diagnosis, 9 skiers and 10 swimmers with no previous diagnosis of asthma, 24 healthy controls	FeNO, spirometry, skin prick test, methacholine challenge, induced sputum	Most results of the skiers were pooled together with the results of the swimmers. Although the results of the skiers were not explicitly analyzed separately, it seemed that there were no significant differences in the levels of inflammatory cells or mediators induced between the asthmatic skiers, healthy skiers and non-athletic controls	Moderate. Only the results of the sputum samples were separately reported in skiers, and there was no statistical analysis

#### Seasonal variation in bronchial reactivity or airway inflammation

Norwegian cross-country skiers and controls were followed for 1 year to assess the seasonal changes in bronchial reactivity and respiratory tract infections during their mandatory military service [40–42]. There were no differences in lung function between the groups or changes in lung function during the study period. Provocative doses in the methacholine test (10% reduction in FEV1) decreased during the winter (i.e., increase in bronchial responsiveness) but increased again towards the summer. The methacholine test was also conducted when a subject had a respiratory infection. Among the skiers, the provocative dose decreased significantly after a respiratory infection, whereas in controls it did not. The provocative dose in skiers did not return to baseline values until six weeks after infection [40–42].

Kennedy et al. 2016 [38] followed eighteen female skiers for one season, performed an analysis of induced sputum and used the Leicester Cough Questionnaire (LCQ). Measurements were conducted three times during the season: at the beginning and end of the training season and during the competition season. No changes in induced sputum cell counts or LCQ were observed between the measurements in the training season, but there was a significant increase in the amounts of lymphocytes and eosinophils in induced sputum from the first to third measurements (0.17 vs. 0.55 × 10^6^ 1/g, 0.014 vs. 0.104 × 10^6^ 1/g, *p* < 0.05) [38].

### Effect of anti-asthmatic treatment in non-asthmatic and asthmatic skiers

There were two studies investigating the effect of bronchodilators on lung function and exercise performance in skiers [43, 44] and one assessing the effect of anti-inflammatory treatment [30].

Sandsund and others 1998 [43] studied salbutamol exclusively in cross-country skiers. Eight male skiers completed two exercise tests in a climatic chamber set to − 15 °C after the inhalation of salbutamol (3 × 400 μg) or a placebo. Three skiers were using anti-asthmatic medication but had not been diagnosed with asthma. The withdrawal of medication before the test was not reported. FEV_1_ increased significantly after the inhalation of salbutamol before the test and was higher during the test compared to the levels with the placebo at all six time points. No significant changes were observed in maximal oxygen uptake or blood lactate levels, and salbutamol did not have performance-enhancing effects.

In 1999, Sue-Chu et al. [44] investigated the effect of salmeterol on physical performance on a treadmill in a climatic chamber in eight healthy male cross-country skiers at − 15 °C. The inhalation of 50 μg salmeterol significantly improved the FEV1 level before, during, and after the exercise test but did not have an effect on the time to exhaustion (392.5 s with salmeterol vs. 395.6 s with the placebo, *p* = 0.84).

Sue-Chu et al. [30] studied the budesonide treatment over a 22-week period in 25 cross-country skiers who had’ski asthma’ (i.e., two or more asthma-like symptoms, including wheezing and abnormal breathlessness or chest tightness upon exertion, at rest, or upon exposure to irritants and BHR to methacholine) and had not used anti-asthmatic medication. They demonstrated no significant improvement in lung function, airway inflammation or tenascin expression.

### Other asthma-related studies in cross-country skiers

There were three studies comparing skiers’ bronchial reactivity in different diagnostic tests [31, 34, 35], one study comparing self-reported symptoms to bronchial hyperresponsiveness [56] and one study reporting the longitudinal follow-up study in three cross-country skiers [45]. The studies are represented in Table 6.

**Table 6:** Other asthma-related studies in cross-country skiers

Year	Author	Subjects	Protocol	Main finding	Risk of bias
2000	Wilber et al. USA [31]	34 biathletes and 14 cross-country skiers participating in Olympic trials	The incidence of EIB in qualified Olympic athletes by spirometry after Olympic trial race (FEV_1_ ≥ 10%)	In the qualifying Olympic team, no biathletes had EIB, 57% of female and 43% of male cross-country skiers had EIB	Moderate. The results are represented as subgroup analysis from qualified Olympic athletes and the number of athletes is not reported
2010	Sue-Chu et al. Norway [35]	58 cross-country skiers (18.1 yrs), 10 skiers with prior asthma diagnosis	Airway hyperresponsiveness to methacholine (PD20 ≤ 1814 μg), AMP (adenosine 5-monophospate, ≤ 50.5 mg), mannitol (≤ 635 mg), 8 min EVH test and 4.7 km field exercise challenge (≥ 10% FEV_1_ decrease at two consecutive time points)	Heterogenous responsiveness to different stimuli among skiers Among 58 skiers40% had positive methacholine test, 9% had positive AMP test, and 5% had positive mannitol test Among 33 skiers 9% had a positive EVH test, and 18% had a positive field exercise challenge	Moderate (the authors own stock)
2007	Stensrud et al, Norway [34]	24 Norwegian national cross-country team skiers	Spirometry after cross-country ski race (≥ 10% FEV_1_), methacholine challenge (PD_20_ ≤ 1600 μg = 8 μmol)	After a ski race 8% of the skiers had bronchial obstruction. 38% had a positive methacholine test	Low
2010	Stenfors, Sweden [56]	46 cross-country skiers or biathletes on national or international level	Multiple self-reported symptoms compared to bronchial hyperresponsiveness in methacholine or mannitol challenge and EVH	Self-reported symptoms had reasonable negative predictive values but very low positive predictive values in relation to bronchial hyperresponsiveness	Low. The sensitivities and specificities of the questions are not analyzed separately in those with and without known asthma or asthma medication
2004	Verges et al. France [45]	One female 19 years, and two male cross-country skiers 21 and 22 years of age and one unreported skier	Follow-up study 9–12 years with intermittent lung function tests, including spirometry and methacholine challenge	Three reported athletes developed objective signs of variable airway obstruction but tests were not systematically positive	Low

In short, three studies [31, 34, 35] assessed the prevalence of BHR among skiers using different tests. There was marked variation in the proportion of subjects having positive test results using different protocols. Stenfors reported that the self-reported symptoms have poor diagnostic accuracy in predicting BHR [56]. The longitudinal case study by Verges and others showed variable signs of airway obstruction in three skiers [45].

### Risk of bias

The risk of bias in the selected articles was assessed by the Cochrane risk of bias tool. Among the 33 articles that were included in the synthesis, 19 articles were considered to have a low risk of bias, 9 articles were considered to have a moderate risk of bias, and 3 articles were considered to have a high risk of bias. Two articles were excluded from the review process due to a very high risk of bias and unclear reporting [65, 66]. See electronic Supplementary File 2 for details.

## Discussion

The aim of this review was to establish an overview of the current literature on asthma in competitive cross-country skiers. Cross-country skiing, nordic combined and biathlon are the only Olympic endurance sports that take place outdoors in possible subfreezing temperatures. A high ventilation rate and large amounts of inhaled cold and dry air leads to extremely high demands on lung function and may cause epithelial damage.

### Prevalence of asthma, asthmatic symptoms and use of asthma medication among skiers

Among cross-country skiers, the mean prevalence of self-reported physician-diagnosed asthma was 21%, and for a combination of self-reported physician-diagnosed asthma and asthma based on lung function measures was 28%, and that for objectively verified asthma was 20% [19–27]. These values are considerably higher than the prevalence of asthma in the general population, which is approximately 10% [4, 5]. Three of the studies also included controls who did not engage in competitive sports, and the prevalence of asthma was significantly higher in cross-country skiers [20, 21, 27].

About 10% of people in the general population are diagnosed with asthma, and it has been estimated that approximately another 5–10% have asthma-like symptoms [6]. According to the current review, the prevalence of at least one asthma-like symptom among competitive cross-country skiers was high and varied between 22 and 84%. However, it is important to note that asthma-like symptoms as such are often not related to asthma [56]. In studies where at least two symptoms were required to be considered asthma-like, the highest prevalence was 51% [22]. In all three studies that compared the difference between cross-country skiers and controls, the prevalence of asthma-like symptoms was higher in skiers [20, 21, 27].

The high prevalence of asthma among competitive skiers is probably not only related to the sport but also cold air exposure and high ventilatory demand, since the prevalence of asthma varies between sports with high and low ventilatory demand [8].

The relative frequency of diagnosed asthma to that of asthmatic symptoms without a formal diagnosis is partly related to the responsibility of health care professionals to suspect asthma and conduct diagnostic testing in symptomatic subjects. If there is a high threshold for suspecting and testing for asthma, asthma may be underdiagnosed, and a high proportion of subjects with asthma suffer from symptoms without proper a diagnosis and medication. The prevalence of asthma-related symptoms relative to the prevalence of asthma differed across the studies included in this review. In three studies, the number of symptomatic skiers was higher than that of asthmatic skiers [21, 22, 25], but in two more recent studies, there were more asthmatic skiers than skiers with asthma-related symptoms without a diagnosis of asthma [26, 27]. Furthermore, in many studies, a large share of previously healthy skiers had variable airway obstruction compatible with asthma, and this proportion was higher in earlier studies and lower in more recent studies [19, 32, 33, 35, 36, 44]. Together, these results suggest that asthma has been underdiagnosed but that this problem has diminished according to the latest studies, possibly due to increased general awareness of asthma among skiers. In most of the studies, the criteria for asthma used with the different lung function tests were based on international guidelines or common practice, but not all criteria were based on these guidelines or common practice (e.g., changes in MMEF after exercise). There are no studies discussing possible overdiagnosis of asthma in cross-country skiers. It is important to note that symptoms as such are not reliable predictors of airway hyperresponsiveness and asthma [56]. In studies reporting self-reported physician-diagnosed asthma it is not clear if objective measures had been used to diagnose asthma or if the diagnosis was based on symptoms only.

The prevalence of asthma medication use was 23% in 1146 skiers across six studies. The proportion of competitive skiers reporting the use of asthma medication is in accordance with the reported prevalence of asthma in this population. Based on these results, it can be cautiously stated that there is no evidence for the misuse of asthma medication in skiers. However, based on all the studies included, it cannot be concluded whether the subjects with a diagnosis of asthma and the subjects using asthma medication were the same subjects. In addition, the use of asthma medication is based on self-reports only. Of the studies in this review, only Heir and Oseid [21] reported the use of asthma medication among skiers without a diagnosis of asthma, and they found that nine percent of athletes with no asthma diagnosis used asthma medication.

The use of asthma medication among competitive skiers has been a controversial topic and widely discussed in media. The use of corticosteroids is now allowed and also the use of several bronchodilators is allowed in up to relatively high doses (salbutamol 1600 ug over 24 h period and 800 ug over 12 h period, formoterol 54 ug over 24 h, salmeterol 200 ug over 24 h) [60]. We identified two studies in which the effects of a relatively high dose of a short-acting β_2_-agonist (salbutamol) or a normal therapeutic dose of a long-acting β_2_-agonist (salmeterol) on lung function and exercise capacity were studied in healthy skiers or skiers with treated asthma [43, 44]. Interestingly, exercise capacity was not affected by these bronchodilators in either of the studies, although lung function was mildly improved, as expected. These results suggest that occasional use of bronchodilators in normal therapeutic doses does not improve exercise capacity, but it is beneficial in preventing bronchoconstriction in skiers with asthma. However, high oral doses of salbutamol or a combination of inhaled β_2_-agonists improved sprinting capacity or maximal strength in two studies [47, 48] acutely and in longitudinal use. It remains to be studied whether these kinds of improvements in strength may benefit healthy cross-country skiers to improve, for example, their final sprint performance. Moreover, there is a lack of research investigating the longitudinal effects of bronchodilators on exercise performance apart from previously mentioned study by Hostrup and others [47].

### Mechanisms of asthma in skiers

There are only a few studies on the cellular and inflammatory mechanisms of asthma among skiers. In general, skiers had higher levels of inflammatory cells in their airways than did healthy non-skiers. Interestingly, inflammatory changes were observed in the airways of both asthmatic and non-asthmatic skiers, and the level of eosinophils was higher in non-skiers with asthma than in skiers with asthma. The exhaled nitric oxide concentration, an inflammatory biomarker associated with eosinophilic inflammation in individuals with asthma, was not different between skiers and healthy controls and was lower in the skiers than in asthmatic controls. Taken together, these results suggest that the distribution of inflammatory endotypes may differ between skiers and non-skiers with asthma that eosinophilic inflammation may not be as prevalent in skiers with asthma and that skiing even in the absence of asthma may trigger noneosinophilic inflammation.

Asthma is usually associated with chronic airway inflammation [1]. Currently, asthma is divided into different endotypes based on the underlying inflammatory mechanisms. The most frequent inflammatory endotypes include allergic eosinophilic asthma, non-allergic eosinophilic asthma, neutrophilic asthma and pauci-granulocytic asthma [49]. In all subjects, whether they have asthma or not, an increase in minute ventilation and dryness of the inhaled air increases the loss of water from epithelial lining fluid (ELF) covering the airway mucosa, making it hypertonic. Mast cells and other inflammatory cells are present in the airways of people with asthma, and they are activated to excrete inflammatory mediators, such as histamine and leukotrienes, in a hypertonic environment. This so-called osmotic mechanism is thought to trigger airway obstruction in asthmatic people during or after exercise [13, 50].

Neutrophilic inflammation in asthma is associated with different irritating stimuli [51], and exposure to cold air is reported to increase airway neutrophilia in people with asthma [52]. Since skiers compete and train in cold conditions and cold air has a very low absolute humidity, their airways are repeatedly exposed to a loss of water and increased osmolality of the epithelial lining fluid. This repeated exposure to high ventilation rates in cold air may, in addition to triggering airway obstruction in people with established asthma, cause irritation and low-grade chronic inflammation and thereby induce asthma in skiers. This idea is also supported by the findings that bronchial hyperresponsiveness and the number of inflammatory cells in airways increase among skiers during the winter [38, 40]. It is important to note that airway infections increased bronchial responsiveness in skiers contrary to healthy controls [41].

The age at onset of asthma is associated with the inflammatory mechanism underlying asthma. Allergic eosinophilic asthma often begins in childhood or early adulthood, while non-allergic eosinophilic and noneosinophilic endotypes more often begin in adulthood [49]. As asthma is a relatively common disease in the general population, some skiers with asthma have likely developed asthma even if they had never started skiing, but some of the skiers may have true skiing-induced asthma. As the onset age of asthma was higher among skiers than among non-skiers with asthma [27] and in one study, none of the skiers reported the onset of asthma in early childhood [19], the prevalence of allergic eosinophilic asthma may be lower among skiers than among non-skiers with asthma. This concept is logical, since children who are diagnosed with allergic asthma during childhood are probably less likely to engage in sports at the competitive level and start an active career as a skier. On the other hand, once young people start actively training and competing in cross-country skiing, they may be at a higher risk of developing asthma due to repeated airway irritation with cold air exposure. The fact that allergic asthma is eosinophilic and asthma induced by irritation is more often neutrophilic can partly explain why asthma in skiers in the existing studies was associated more strongly with neutrophilic than eosinophilic inflammation. The relatively higher neutrophilic activity in skiers with asthma may also have treatment-related consequences, since anti-inflammatory treatment with inhaled glucocorticoids is most effective in people with asthma and eosinophilic inflammation [53–55]. In fact, this concept is in line with the results of a study that assessed the effect of inhaled budesonide on ski asthma and found no improvement in lung function or airway inflammation [30].

### Detecting asthma in skiers

Due to the high prevalence of asthma among competitive skiers, asthma should be actively screened. Since self-reported symptoms are not a very reliable predictor of asthma-like reversible or variable airway obstruction among skiers [56], objective measures of lung function are needed. In general, tests of bronchial hyperresponsiveness seem to be more sensitive tools in detecting asthma than spirometry with bronchodilation. Based on two studies [34, 35], the tests used to detect hyperresponsiveness in order of the highest to the lowest level of sensitivity are as follows: the methacholine challenge, exercise test, EVH test, AMP challenge and mannitol challenge. Of these tests, the methacholine test acts directly on muscarinic receptors in bronchial smooth muscle, whereas the other tests are indirect and cause airway obstruction by increasing the tonicity of epithelial lining fluid, which triggers inflammatory cells to release mediators that activate smooth muscle [2]. It is understandable that direct tests of hyperresponsiveness, such as the methacholine challenge, are more sensitive than indirect tests, because the latter tests are dependent on the presence and activity of inflammatory cells in airways. However, it is important to note that the sensitivity and specificity of each of these tests is dependent on the threshold used (e.g., the provocative dose of methacholine or the level of decrease in FEV_1_ in exercise challenge). EVH test is usually considered more sensitive than the exercise test in detecting hyperresponsiveness [57]. The opposite finding in skiers in one study may be related to the fact that top athletes may be able to achieve even higher minute ventilation in their own sport than in the EVH test with a predefined level of minute ventilation.

The onset and severity of asthma-related symptoms may progress gradually, and athletes may not be able to differentiate asthma-related symptoms from normal breathlessness and exhaustion. Therefore, it may be useful to conduct regular screenings of asthma to promote athletes’ health. This would decrease the probability of athletes training and competing with asthma that they are not aware of. On the other hand, regular screening of asthma requires resources and there is no universal agreement over the correct criteria of asthma in athletes. A single positive test result in a direct bronchial challenge with no asthma-related symptoms may not indicate asthma and needs further verification in indirect challenges, since healthy people, especially cross-country skiers, may have increased bronchial responsiveness during the winter season, as shown in the study by Heir [40]. A positive test result in an indirect bronchial challenge, such as the exercise challenge, with no asthma-related symptoms may, however, be indicative of asthma, because people have a measurable level of obstruction in real-life conditions, which affect exercise performance. Regardless of whether the lung function test is used, it is worth considering that due to the lack of a gold standard in asthma diagnostics, all the diagnostic cut-off values are somewhat controversial and are based on studies in non-athletes. Whether different cut-off values are needed for skiers or other competitive athletes is not known.

Exercise-induced laryngeal obstruction (EILO) is a transient, reversible and inappropriate narrowing of the larynx in response to exercise [58]. In contrast to exercise-induced asthma, the symptoms in EILO peak during exercise, whereas the symptoms in exercise-induced asthma may worsen after exercise. There is one study assessing the prevalence of EILO in a retrospective cohort by Nielsen and others [61]. EILO was found in 35.2% (*n* = 31) of the studied athletes and asthma was concurrently present in 38.7% (*n* = 12) in athletes with EILO. Since asthma and laryngeal obstruction may coexist [59, 61], it is important to remember that exercise-induced symptoms in an individual treated for asthma may not be due to bronchial obstruction but rather due to untreated laryngeal problems.

### Strengths and limitations of the review

This review is based on a systematic literature search focusing on all aspects of asthma in competitive cross-country skiers, and all the major databases were searched without restrictions. The number of studies identified and included was relatively small, and each topic was often covered by only a few studies with considerable differences in aspects of the studies, such as the methods, definitions, and cut-off levels. One common problem among skiers and a possible confounder in all asthma-related research is repeated respiratory infections. Intensive training in cold conditions, a high level of stress frequent travelling and mass gatherings in sports increase the risk for infection and symptoms suggestive of asthma. In fact, one of the studies that assessed bronchial responsiveness among skiers reported increased bronchial responsiveness after an airway infection in skiers but not in controls [41].

Studies included in the review were published between 1993 and 2018. During this time, the understanding of asthma pathophysiology has increased, and the diagnostic methods and criteria have been standardized. Moreover, the treatment of asthma and doping regulations have evolved based on new knowledge. Thus, the methods and research questions have also evolved during this time period.

In our risk of bias assessment, 10% of the articles were considered to have moderate or high risk of bias, and this result should also be taken into account.

The meta-analysis was based on the eight available publications on the prevalence of asthma and use of asthma medication. The relatively small number of published studies decreases the reliability of the results and makes it challenging to use formal publication bias assessment. The methods assessing possible publication bias usually require more than ten articles to be included in the meta-analysis due to heterogeneity and low power [64]. We used random effects model in the meta-analysis. However, the number of studies was small and this may affect the reliability of significance testing, as the small number of studies undermines the accuracy of the estimate of the between-study variance, standard error of the estimated prevalences and thereby ultimately the significance testing.

## Conclusions and future directions

Based on the current literature, asthma and asthma-like symptoms are highly prevalent among competitive cross-country skiers. In lung function tests many previously healthy skiers have bronchial hyperresponsiveness compatible with asthma, i.e., asthma is possibly underdiagnosed in this population. Systematic screening for asthma with objective tests for bronchial hyperresponsiveness is thus warranted in competitive skiers. There is also a need for international consensus over the criteria of asthma and its treatment in athletes. The self-reported use of asthma medication is not more frequent than the diagnosis of asthma among skiers, suggesting that there is currently no remarkable overuse of asthma medication in this group. The mechanisms of asthma is more often related to neutrophilic inflammation in skiers than in other people with asthma, and this result may be related to a lower prevalence of early-onset allergic asthma (negative selection since small children with asthma are less likely begin an active skiing career) and neutrophilic inflammation induced by repetitive exposure to cold dry air and a high ventilatory demand. According to one study, there is an indication that inhaled glucocorticoids may not be as effective in treating skiers’ asthma as it is in treating asthma in general, and this result is in accordance with the previous conclusion that inhaled corticosteroids are most effective in treating eosinophilic asthma.

Future research should focus on the role of the intensity of training and repeated airway infections on the development of asthma among competitive skiers. The optimal treatment, current asthma control and natural course of asthma are not known in this population. Currently, there is also no information on how asthma among competitive skiers affects their ability to train, participate in competitions and succeed in their career.

## Electronic supplementary material

Below is the link to the electronic supplementary material.

Supplementary file1 (PDF 79 kb)

Supplementary file2 (PDF 83 kb)

Supplementary file3 (PDF 98 kb)

## Abbreviations

EIB: Exercise-induced bronchoconstriction
EVH: Eucapnic voluntary hyperpnoea
AMP: Adenosine 5´-monophosphate
MMEF: Maximal mid-expiratory flow
