# Supplementary material for: Asthma in Competitive Cross-Country Skiers: A Systematic Review and Meta-analysis
Source: Sports Med. 2020 Sep 11;50(11):1963–81. doi: 10.1007/s40279-020-01334-4 (PMC7575483; doi:10.1007/s40279-020-01334-4)
Supplement: Supplementary file 1 — Supplementary file1 (PDF 79 kb) [file 40279_2020_1334_MOESM1_ESM.pdf]

Asthma in competitive cross-country skiers – A systematic review and meta-analysis
Sports Medicine

Mäki-Heikkilä, Rikhard[1], Karjalainen, Jussi[1,2], Parkkari, Jari[3], Valtonen, Maarit[4], Lehtimäki, Lauri[1,2]

[1]Faculty of Medicine and Health Technology, Tampere University, Tampere, Finland; [2]Allergy Centre, Tampere University Hospital, Tampere, Finland; [3]Tampere Research Center of Sports Medicine, UKK Institute, Tampere, Finland; [4]KIHU – Research Institute for Olympic Sports, Jyväskylä, Finland

Corresponding author: Lauri Lehtimäki, lauri.lehtimaki@tuni.fi

Supplemental File 1. Search strategy and search words used.

Search date: August 29, 2019.

Search engines: PubMed, EBSCO Academic Search Premier, Web of Science, Scopus, Cochrane Library and clinicaltrials.gov

In PubMed, MeSH –terms asthma, bronchoconstriction, hypersensitivity, dyspnea, cough, sputum, bronchial spasm, bronchial hyperreactivity, cold temperature, respiratory function test, bronchial provocation test, spirometry, anti-asthmatic agents, bronchodilator agents, cholinergic antagonists, adrenergic beta-2 receptor agonists, muscarinic antagonists, glucocorticoids, chromones, leukotriene antagonists, ipratropium, oxitropium, tiotropium, umeclidinium, acridinium, glycopyrronium, albuterol, terbutaline, formeterol, salmeterol, vilanterol, indacaterol, olodaterol, beclomethasone, fluticasone, ciclesonide, budesonide, flunisolide, mometasone, montelukast, zafirlukast and skiing were included in the search.

Search terms were divided into two fields.

(asthma* OR EIA OR EIB OR bronchoconstriction OR hypersensitivity OR hyperresponsiveness OR "airway inflammation*" OR "bronchial hyperresponsiveness" OR BHR OR dyspnea OR cough OR sputum OR irritation OR stridor OR "airway symptom*" OR bronchospasm* OR "bronchial spasm*" OR "bronchial hyperreactivity" OR "cold temperature" OR "respiratory function test*" OR "bronchial provocation test*" OR "spirometry" OR "methacholine challenge*" OR "eucapnic voluntary ventilation test*" OR "eucapnic voluntary hyperpnoea" OR EVH OR "anti-asthmatic agent*" OR "bronchodilator agent*" OR "cholinergic antagonist*" OR "adrenergic beta-2 receptor agonist*" OR "muscarinic antagonist*" OR glucocorticoid* OR theophylline OR chromone* OR "leukotriene antagonist*" OR "anti-asthmatic drug*" OR "anti-asthmatic medication*" OR "asthma medication*" OR "asthma drug*" OR "anti-asthmatic agents" OR "bronchodilator agents" OR "cholinergic antagonists" OR "adrenergic beta-2 receptor agonists" OR "muscarinic antagonists" OR glucocorticoids OR chromones OR "leukotriene antagonists" OR ipratropium OR oxitropium OR tiotropium OR umeclidinium OR acridinium OR glycopyrronium OR albuterol OR salbutamol OR terbutaline OR formoterol OR salmeterol OR vilanterol OR indacaterol OR olodaterol OR beclomethasone OR fluticasone OR ciclesonide OR budesonide OR flunisolide OR mometasone OR montelukast OR zafirlukast)
AND
(skiing OR "cross-country ski*" OR skier* OR "cold weather athlete*" OR "winter athlete*" OR "winter sport*" OR "nordic skiing" OR biathlon OR biathlete* OR "nordic combined" OR "endurance sport*" OR "ski-orienteer*")
