# Supplementary material for: Asthma in Competitive Cross-Country Skiers: A Systematic Review and Meta-analysis
Source: Sports Med. 2020 Sep 11;50(11):1963–81. doi: 10.1007/s40279-020-01334-4 (PMC7575483; doi:10.1007/s40279-020-01334-4)
Supplement: Supplementary file 2 — Supplementary file2 (PDF 83 kb) [file 40279_2020_1334_MOESM2_ESM.pdf]

Asthma in competitive cross-country skiers – A systematic review and meta-analysis
Sports Medicine

Mäki-Heikkilä, Rikhard (1), Karjalainen, Jussi (1,2), Parkkari, Jari (3), Valtonen, Maarit (4), Lehtimäki, Lauri (1,2)
(1) Faculty of Medicine and Health Technology, Tampere University, Tampere, Finland; (2) Allergy Centre, Tampere University Hospital, Tampere, Finland; (3) Tampere Research Center of Sports Medicine, UKK Institute, Tampere, Finland; (4) KIHU – Research Institute for Olympic Sports, Jyväskylä, Finland
Corresponding author: Lauri Lehtimäki, lauri.lehtimaki@tuni.fi

Supplemental file 2: Risk of bias assessments

Study	year published	Title	Risk of bias<br>Randomisation sequence generation: was the allocation sequence adequately generated? OR were subjects recruited properly?	Treatment allocation concealment: was the allocated treatment adequately concealed from study participants and clinicians and other healthcare or research staff at the enrolment stage?	Blinding: were the personnel assessing outcomes and analysing data sufficiently blinded to the intervention allocation throughout the trial?	Completeness of outcome data: were participant exclusions, attrition and incomplete outcome data adequately addressed in the published report?	Selective outcome reporting: is there evidence of selective outcome reporting and might this have affected the study results?	Other sources of bias: was the trial apparently free of any other problems that could produce a high risk of bias?	Overall bias: low, moderate, high, excluded	LOW<br>18	MODERATE<br>10	HIGH<br>3	VERY HIGH<br>2	TOTAL<br>33
Larsson et al. 1993	1993	High prevalence of asthma in cross country skiers	Recruiting by invitation from ski clubs in Sweden	cross-sectional study	no blinding	42/47 of the invited participated.	no evidence	Skiers with ICS medication did not dicontinue their medication for tests. Unidentical methacholine test protocols in study centers.	Moderate. Representativeness of subject to whole skier population and recruitment methods. Different methacholine challenge in different locations.		1			
Larsson et al. 1994	1994	Self-reported obstructive airway symptoms are common in young cross-country skiers	no randomisation in skiers, but almost the best skiers included. controls selected from same schools as skiers	not applicable, cross-sectional study	no blinding	Response rate high: 98 % in skiers, 90 % controls	The aim of the study was to estimate the prevalence of self-reported airway symptoms and asthma as well as the use of anti-asthmatic medication. Results clearly reported	no evidence	Low	1				
Heir and Oseid 1994	1994	Self-reported asthma and exercise-induced asthma symptoms in high-level competitive cross-country skiers	no randomisation in skiers, but almost the best skiers included. controls selected to match skiers' demography	not applicable, cross-sectional study	no blinding	Response rate very high: 100 % in skiers, 79 % controls	Aim of the study was prevalence of asthma and results clearly stated	no evidence	Low	1				
Sue-Chu et al. 1996	1996	Prevalence of asthma in young cross-country skiers in central Scandinavia: Differences between Norway and Sweden	No randomisation. Not clear if all skiers eligible in the region were invited	observational study, no treatment allocated	no blinding	100 % completed the study.	no evidence	no control group who don't participate in skiing. No information about the validity of questionnaire, validity of total prevalence of asthma, if diagnosis was self-reported	Low	1				
Michalak et al. 2002	2002	Prevalence of asthma in athletes, influence of sport and environmental exposure (French)	no randomisation	cross-sectional study	no blinding	all completed	no evidence	no evidence	Low.	1				
Langdeau et al. 2004	2004	Comparative prevalence of asthma in different groups of athletes: a survey.	no randomisation, questionnaire sent to all eligible participants in Quebec area	not applicable, cross-sectional study	no blinding	48 % responded, percentage of responded skiers not reported. other disciplines as comparison/controls	data from different disciplines mixed: only prevalence of asthma could be extracted	valid question pattern from ECHRS questionnaire	Moderate. Small sample size		1			
Turmel et al. 2012	2012	Cardiorespiratory screening in elite endurance sports athletes - the quebec study	no randomisation	screening study, no treatment allocated	no blinding	133/375 invited participated. Selection bias? number of excluded subjects not reported. criteria stated	no evidence	Possible selection bias	Moderate. Possible selection bias		1			
Norqvist et al. 2015	2015	Self-reported physician-diagnosed asthma among swedish adolescent, adult and former elite endurance athletes	no randomisation	postal questionnaire, no treatment allocated	no blinding	response rate 82 %	no evidence	selection bias: those with problems respond. recall bias of onset of asthma: not likely.	Low.	1				
Eklund et al. 2018	2018	Prevalence, age at onset, and risk factors of self-reported asthma among swedish adolescent elite cross-country skiers.	no randomisation	postal questionnaire, no treatment allocated	no blinding	response rate 96 % in skiers, 48 % in controls.	no evidence	Low response rate in controls. Even if true prevalence of asthma in controls is lower, the difference is still significant.	Low.	1				
Heir 1994	1994	Longitudinal variations in bronchial responsiveness in cross-country skiers and control subjects	It is not certain if all skiers and controls in conscript service were included. Skiers in conscript service are not an appropriate selection to represent elite skiers	not applicable, longitudinal study	no blinding	19/22 skiers and 22/24 controls completed, reasons clearly stated	BHR variation investigated and it is reported clearly.	Methacholine cutoff criteria different from current protocol (PC10 dose, FEV1 fall ≥ 10 %)	Moderate. Representativeness of the subjects as elite skiers		1			
Heir and Larsen 1995	1995	The influence of training intensity, airway infections and environmental conditions on seasonal variations in bronchial responsiveness in cross-country skiers	No randomisation. Same cohort as Heir et al. 1995	observational study, no treatment allocated	no blinding	All subjects completed the study.	no evidence	Military service differs from general life in skiers. Effect of environment? Reliability of training diaries?	Moderate. Reliability to conclude that highly intensive training causes BR to increase.		1			
Heir et al. 1995	1995	Respiratory tract infection and bronchial responsiveness in elite athletes and sedentary control subjects	No randomisation. Recruitment pool 19 skiers and 12 contracted an infection. Controls 22 and 10 contracted an infection from the same military camp.	observational study, no treatment allocated	no blinding	No participant exclusion. Aim of the study and the result clearly reported	no evidence	diverse causes for infection. Cause for infection not reported in 14/22 subjects. Could the results be applied to skiers in general?	Low.	1				
Sue-Chu et al. 1998	1998	Lymphoid aggregates in endobronchial biopsies from young elite cross-country skiers.	no randomisation	laboratory study, no treatment allocated	no blinding	100 %	none	Controls statistically significantly older (24,1 vs. 17,6 yr), effect?	Low	1				
Sandsund et al. 1998	1998	Effect of cold exposure (-15°C) and Salbutamol treatment on physical performance in elite nonasthmatic cross-country skiers	Test order randomised. Recruitment methods not reported	observational study, no treatment allocated	salbutamol administration double-blinded	100 % completed the study.	aim of the study and outcomes match	Three skiers used previously anti-asthmatic medication but exact medication or withhold period not reported. Running as the exercise modality, not skiing. Probably not relevant.	Low.	1				
Sue-Chu et al. 1999	1999	Non-invasive evaluation of lower airway inflammation in hyper-responsive elite cross-country skiers and asthmatics.	no randomisation	test study, no treatment conducted	no blinding	no report of exclusion: 100 % completed the study	none	none observed	Low	1				
Sue-Chu et al. 1999	1999	Bronchoscopy and bronchoalveolar lavage findings in cross-country skiers with and without "ski asthma".	no randomisation	test study, no treatment conducted	no blinding of the sample assessment	100 %	none	sample assessment not blinded	Low	1				
Sue-Chu et al. 1999	1999	Salmeterol and physical performance at - 15°C in highly trained nonasthmatic cross-country skiers	test order randomised. recruitment methods not reported	cross-over design.	double-blind, block-randomised, placebo-controlled, cross-over study design	100 % of the subjects completed	no evidence	Running as the exercise modality, not skiing. Probably not relevant.	Low	1				

Supplemental file 2: Risk of bias assessments

			Risk of bias							LOW	MODERATE	HIGH	VERY HIGH	TOTAL
										18	10	3	2	33
**Study**	**year published**	**Title**	**Randomisation sequence generation: was the allocation sequence adequately generated? OR were subjects recruited properly?**	**Treatment allocation concealment: was the allocated treatment adequately concealed from study participants and clinicians and other healthcare or research staff at the enrolment stage?**	**Blinding: were the personnel assessing outcomes and analysing data sufficiently blinded to the intervention allocation throughout the trial?**	**Completeness of outcome data: were participant exclusions, attrition and incomplete outcome data adequately addressed in the published report?**	**Selective outcome reporting: is there evidence of selective outcome reporting and might this have affected the study results?**	**Other sources of bias: was the trial apparently free of any other problems that could produce a high risk of bias?**	**Overall bias: low, moderate, high, excluded**					
Karjalainen et al. 2000	2000	Evidence of airway inflammation and remodeling in ski athletes with and without bronchial hyperresponsiveness to methacholine.	no randomisation	test study, no treatment conducted	no	100 %	no evidence	asthmatic status of the skiers? Confounding factor	Low	1				
Sue-Chu et al. 2000	2000	Placebo-controlled study of inhaled budesonide on indices of airway inflammation in bronchoalveolar lavage fluid and bronchial biopsies in cross-country skiers.	randomisation of treatment	treatment randomised at the beginning.	double-blinded drug treatment	one skier withdrew from study	No evidence of selection bias	two different observers analysising the BAL samples. One in Trondheim, one in Helsinki	Low			1		
Wilber et al. 2000	2000	Incidence of exercise-induced bronchospasm in Olympic winter sport athletes.	no randomisation. initial pool of subjects from participants in Olympic trials. inclusion criteria: Olympic team 1998	cross-sectional study	no blinding	3 % of the olympic team could not be tested (all disciplines, not clear which sport)	total number of subjects in the olympic team not reported.	Selection bias	High. total number of subjects in the olympic team was not reported.	1				
Ogston and Butcher 2002	2002	A Sport-Specific Protocol for Diagnosing Exercise-Induced Asthma in Cross-Country Skiers	no randomisation	sport-specific diagnostic test. no treatment allocated	no blinding	99/101, two excluded because of acute respiratory illness	No evidence	none observed	Low		1			
Pohjantähti et al. 2005	2005	Exercise-induced bronchospasm among healthy elite cross country skiers and non-athletic students	no randomisation	sport-specific diagnostic test. no treatment allocated	no blinding	87 %, 3 excluded because of prior asthma diagnosis. all other subjects completed the study	no evidence	only study to use ≥ 20 % decrease in MMEf as one criteria for asthma diagnosis. EIB and asthma was diagnosed also based on changes in MMEF after exercise test, which is not a validated criterion	Moderate. MMEF is not in international diagnostic guidelines.	1				
Stensrud et al. 2007	2007	Bronchial hyperresponsiveness in skiers: field test versus methacholine provocation?	no randomisation	diagnostic test, no treatment allocated	no blinding	100 %	no evidence		Low		1			
Sue-Chu et al. 2010	2010	Airway hyperresponsiveness to methacholine, adenosine 5-monophosphate, mannitol, eucapnic voluntary hyperpnoea and field exercise challenge in elite cross-country skiers.	test order randomised	diagnostic test, no treatment allocated	no blinding	100% for the first part, 57 % for second (2 personal reasons, 23 respiratory illness)	no evidence	GlaxoSmithKline funded the study. Some authors receive royalty for mannitol tests and own Pharmaxis stock. Pharmaxis produces Aridol which may have been used but not mentioned	Moderate	1				
Dickinson et al. 2011	2011	Diagnosis of exercise-induced bronchoconstriction: eucapnic voluntary hyperpnoea challenges identify previously undiagnosed elite athletes with exercise-induced bronchoconstriction	Recruiting by invitation. Athletes with no prior bronchodilatation test or bronchoprovocation. 100 % participation rate	test study, no treatment conducted	no blinding	100 % completed the challenge.	Biathletes mixed with other disciplines, not all data could be extracted.	small sample size.	Low. Small sample size			1		
Zebrowska et al. 2015	2015	Endurance training and the risk of bronchial asthma in female cross-country skiers.	no randomisation	screening study, no treatment allocated	no blinding	spirometry values not shown, only percentages	no results after exercise challenge. would have been possible but not reported if conducted	no repeated measurements of NO-levels. Time of day not reported (possibly after blood samples).	High. Selective reporting and measurement times not reported. Possible asthma medication use not reported		1			
Kennedy et al. 2016	2016	Airway inflammation, cough and athlete quality of life in elite female cross-country skiers: A longitudinal study	Recruiting from Canadian national and other top teams. Possible refusals of eligible athletes in recruiting process not reported. no controls	longitudinal study	no blinding	100 % of the recruited completed the challenge.	10/18 provided sputum induction samples, reason not reported	no controls, generalization to all elite athletes, including men	Moderate. Sputum sample exclusions not reported				1	
Nikitina et al. 2013	2013	The interaction between respiratory function and exhaled nitric oxide in exercise-induced bronchoconstriction in sportsmen	no randomisation	test study, no treatment allocated. Exercise protocols and intensities not fully reported	no blinding	number of athletes with and without EIB not reported.	confusing terminology: sportsmen and sportswomen. discussion only about sportsmen.	number of athletes in different groups not reported. Not clear if "sportsmen" in results also concerned women	Very high, excluded				1	
Nikitina 2014	2014	Efficacy of antileukotriene therapy in exercise-induced bronchospasm in skiers and biathlonists	no randomisation	only athletes with EIB after the first test received montelukast. no placebo used. protocol for testing EIB not clearly reported.	no blinding	How many athletes did not have anymore FEV1 ≥ 10 % decrease after drug therapy? How many athletes conducted tests, 10 or 11 athletes?	Where are athletes with no EIB after the first test? Did they do another test 10 days later?	number of athletes in different groups not reported.	Very high, excluded			1		
Stang et al. 2018	2018	The Role of Airway Inflammation and Bronchial Hyperresponsiveness in Athlete's Asthma.	no randomisation. Recruitment criteria ok	no treatment conducted, cross-sectional study	cell count calculation blinded and conducted by two investigators (not reported if individually)	21 % of the original subjects excluded due inadequate sputum sample	no evidence	7/20 of the asthmatic athletes used ICS. Test conducted all year round, including in the winter when the stress on the airways is maximum in skiers.	Moderate	1				
Stenfors 2010	2010	Self-reported symptoms and bronchial hyperresponsiveness in elite cross-country skiers	no randomisation	test study, no treatment conducted	no blinding	100 %	no evidence	Proportion of subjects with asthma or use of asthma medication not reported	Low. The sensitivities and specificities of the questions are not analysed separately in those with and without known asthma or asthma medication.	1				
Verges et al. 2004	2004	A 10-year follow-up study of pulmonary function in symptomatic elite cross-country skiers – athletes and bronchial dysfunctions	case report	no treatment	no blinding	all cases reported	no evidence	none observed	Low		1			
Rundell et al. 2001	2001	Self-reported symptoms and exercise-induced asthma in the elite athlete.	no randomisation. recruitment methods not clearly reported	cross-sectional study	no blinding	All completed	not all results from different sports reported. athletes regarded as one group.	no evidence	Moderate. Unclear recruitment strategy. Only provides evidence from asthma-related symptoms in skiers, nothing else.		1			
