# Supplementary material for: Asthma in Competitive Cross-Country Skiers: A Systematic Review and Meta-analysis
Source: Sports Med. 2020 Sep 11;50(11):1963–81. doi: 10.1007/s40279-020-01334-4 (PMC7575483; doi:10.1007/s40279-020-01334-4)
Supplement: Supplementary file 3 — Supplementary file3 (PDF 98 kb) [file 40279_2020_1334_MOESM3_ESM.pdf]

Asthma in competitive cross-country skiers – A systematic review and meta-analysis
Sports Medicine

Mäki-Heikkilä, Rikhard (1), Karjalainen, Jussi (1,2), Parkkari, Jari (3), Valtonen, Maarit (4), Lehtimäki, Lauri (1,2)
(1) Faculty of Medicine and Health Technology, Tampere University, Tampere, Finland; (2) Allergy Centre, Tampere University Hospital, Tampere, Finland; (3) Tampere Research Center of Sports Medicine, UKK Institute, Tampere, Finland; (4) KIHU – Research Institute for Olympic Sports, Jyväskylä, Finland

Corresponding author: Lauri Lehtimäki, lauri.lehtimaki@tuni.fi

Supplemental file 3. List of articles included in full-text search
Total: 163 articles, 130 articles excluded, 33 articles included in the review

Author and year	Title	Decision
Accad 1997	Dyspnea, cyanosis, and pulmonary infiltrates in a young skier.	not asthma
Alaranta 2008	Use of prescription drugs in athletes.	review
Alaranta ym. 2004	Asthma medication in Finnish olympic athletes: no signs of inhaled beta2-agonist overuse.	skiers cannot be separated from other subjects
Andersen 2009	Effects of acute oral administration of 4 mg salbutamol on exercise performance in non-asthmatic elite athletes	skiers cannot be separated from other subjects
Anderson 2003	Responses to bronchial challenge submitted for approval to use inhaled beta(2)-agonists before an event at the 2002 Winter Olympics	skiers cannot be separated from other subjects
Backer 2010	Not all who wheeze have asthma	review
Bellon 2004	Asthma among elite athletes	review
Bjermer 1996	Obstructive symptoms in athletes: is it asthma and what to do about it?	editorial, commentary, interview
Blume et al. 2018	Training load, immune status, and clinical outcomes in young athletes: A controlled, prospective, longitudinal study	skiers cannot be separated from other subjects
Bolger 2011	Effect of inspired air conditions on exercise-induced bronchoconstriction and urinary CC16 levels in athletes.	subjects not competitive skiers
Bonini 2015	Asthma, allergy and the olympics: A 12-year survey in elite athletes	skiers cannot be separated from other subjects
Bonsignore 2007	Airways inflammation and sport	review
Bordeleau 2013	Effect Of Ipratropium On Exercise-Induced Cough In Winter Athletes	conference abstract
Bordeleau 2014	Effects of ipratropium on exercise-induced cough in winter athletes: a hypothesis-generating study.	not asthma
Bosco 1998	Testosterone and cortisol levels in blood of male sprinters, soccer players and cross-country skiers	not asthma
Bougault 2008	Airway Hyperresponsiveness (AHR) and Markers of Inflammation or Remodelling in Swimmers and Winter athletes	conference abstract
Bougault 2009	Asthma, airway inflammation and epithelial damage in swimmers and cold-air athletes.	skiers cannot be separated from other subjects
Bougault 2010	Bronchial challenges and respiratory symptoms in elite swimmers and winter sport athletes: Airway hyperresponsiveness in asthma: its measurement and clinical significance.	skiers cannot be separated from other subjects
Bougault 2010	Bronchial Challenges and Respiratory Symptoms in Elite Swimmers and Winter Sport Athletes.	skiers cannot be separated from other subjects
Boulet 2015	Asthma and exercise-induced bronchoconstriction in athletes	review
Boulet et al. 2017	Athletes Do Not Condition Inspired Air More Effectively than Nonathletes during Hyperpnea	subjects not competitive skiers
Butcher 2006	Exercise-induced asthma in the competitive cold weather athlete.	review
Byrne-Quinn 1971	Ventilatory control in the athlete.	skiers cannot be separated from other subjects
Carlsen 1997	The effect of inhaled salbutamol and salmeterol on lung function and endurance performance in healthy well-trained athletes	skiers cannot be separated from other subjects
Carlsen 2001	Can asthma treatment in sports be doping? The effect of the rapid onset, long-acting inhaled beta(2)-agonist formoterol upon endurance performance in healthy well-trained athletes	skiers cannot be separated from other subjects
Carlsen 2012	Sports in extreme conditions: the impact of exercise in cold temperatures on asthma and bronchial hyper-responsiveness in athletes.	review
Carlsen 2012	Mechanisms of asthma development in elite athletes	not asthma
Chalkias 2013	Airway Remodeling and Cardiac Arrest in Long-Distance Ski Races	editorial, commentary, interview
Couillard 2014	Perception of bronchoconstriction following methacholine and eucapnic voluntary hyperpnea challenges in elite athletes	skiers cannot be separated from other subjects

Author and year	Title	Decision
Couto 2015	Two distinct phenotypes of asthma in elite athletes identified by latent class analysis.	skiers cannot be separated from other subjects
Dahlqvist 1992	Exposure to Ski-Wax Smoke and Health Effects in Ski Waxers	subjects not competitive skiers
Derman 2016	The incidence and patterns of illness at the Sochi 2014 Winter Paralympic Games: A prospective cohort study of 6564 athlete days	not asthma
Dickinson 2005	Impact of changes in the IOC-MC asthma criteria: A British perspective	subjects not competitive skiers
Dickinson 2006	Screening elite winter athletes for exercise induced asthma: a comparison of three challenge methods.	skiers cannot be separated from other subjects
Dickinson 2014	The ergogenic effect of long-term use of high dose salbutamol	subjects not competitive skiers
Dickinson et al. 2018	Nonpharmacologic Strategies to Manage Exercise-Induced Bronchoconstriction	review
Dickinson ym. 2011	Diagnosis of exercise-induced bronchoconstriction: Eucapnic voluntary hyperpnoea challenges identify previously undiagnosed elite athletes with exercise-induced bronchoconstriction	**included in the review**
Doyon 2001	Field testing of V̇O2 peak in cross-country skiers with portable breath-by-breath system	equipment validation study
Driessen 2014	Exercise-induced airway obstruction in the cold	review
Engebretsen 2010	Sports injuries and illnesses during the winter olympic games 2010	not asthma
Eriksson et al. 2018	Prevalence, age at onset, and risk factors of self-reported asthma among Swedish adolescent elite cross-country skiers.	**included in the review**
Evans 2005	Cold air inhalation does not affect the severity of EIB after exercise or eucapnic voluntary hyperventilation	subjects not competitive skiers
Fitch 2012	An overview of asthma and airway hyper-responsiveness in Olympic athletes	review
Freberg 2016	Pulmonary function and serum pneumoproteins in professional ski waxers	subjects not competitive skiers
Gonzalez-Millan et al. 2017	Physiological Demands of Elite Cross-Country Skiing During a Real Competition.	not asthma
Haahtela 2008	Mechanisms of asthma in Olympic athletes--practical implications.	review
Heir 1994	Longitudinal variations in bronchial responsiveness in cross-country skiers and control subjects	**included in the review**
Heir 1994	Self-reported asthma and exercise-induced asthma symptoms in high-level competitive cross-country skiers	**included in the review**
Heir 1995	Respiratory tract infection and bronchial responsiveness in elite athletes and sedentary control subjects	**included in the review**
Heir 1995	Salbutamol and high-intensity treadmill running in nonasthmatic highly conditioned athletes	skiers cannot be separated from other subjects
Heir 1995	The influence of training intensity, airway infections and environmental conditions on seasonal variations in bronchial responsiveness in cross-country skiers	**included in the review**
Helenius ym. 1998	Asthma and increased bronchial responsiveness in elite athletes: atopy and sport event as risk factors.	subjects not competitive skiers
Hinde 2017	The effect of temperature, gradient, and load carriage on oxygen consumption, posture, and gait characteristics	subjects not competitive skiers
Hoffman ym. 1997	Acute effects of ski waxing on pulmonary function.	subjects not competitive skiers
Holmberg ym. 2007	Lung function, arterial saturation and oxygen uptake in elite cross country skiers: influence of exercise mode	not asthma
Hoshino 2015	Effect of inhaled corticosteroids on bronchial asthma in Japanese athletes	skiers cannot be separated from other subjects
Iikura 1977	Ski Camp for Asthmatic Children	subjects not competitive skiers
KARJALAINEN 1995	NONSPECIFIC BRONCHIAL HYPERREACTIVITY IN SKI ATHLETES IS ASSOCIATED WITH INCREASED TENASCIN EXPRESSION IN THE BRONCHIAL BASEMENT-MEMBRANE ZONE	conference abstract
Karjalainen ym. 2000	Evidence of airway inflammation and remodeling in ski athletes with and without bronchial hyperresponsiveness to methacholine.	**included in the review**
Kennedy 2016	Airway inflammation, cough and athlete quality of life in elite female cross-country skiers: A longitudinal study	**included in the review**
Kennedy et al. 2018	Respiratory function and symptoms post cold air exercise in female high and low ventilation sport athletes	skiers cannot be separated from other subjects
Kippelen 2012	Respiratory health of elite athletes - preventing airway injury: a critical review.	review

Author and year	Title	Decision
Knopfli ym. 1992	[Acute deterioration of the CO diffusion capacity following exposure to ski-wax vapors].	not asthma
Koskela 2007	Cold air-provoked respiratory symptoms: The mechanisms and management	review
Kotaniemi 2003	Does living in a cold climate or recreational skiing increase the risk for obstructive respiratory diseases or symptoms?	subjects not competitive skiers
Kujala ym. 1996	Asthma and other pulmonary diseases in former elite athletes.	subjects not competitive skiers
Laitinen 1994	[Ski racing and asthma].	editorial, commentary, interview
Langdeau 2004	Comparative prevalence of asthma in different groups of athletes: a survey.	**included in the review**
Larsson 1993	[Does competitive skiing cause asthma? Cold air is suspected to be the primary cause. Interview by Birgit Wilhelmson].	editorial, commentary, interview
Larsson 1993	High prevalence of asthma in cross country skiers.	**included in the review**
Larsson 1994	Self-reported obstructive airway symptoms are common in young cross-country skiers	**included in the review**
Larsson 1997	Influence of a beta2-agonist on physical performance at low temperature in elite athletes.	skiers cannot be separated from other subjects
Larsson 1998	Inhalation of cold air increases the number of inflammatory cells in the lungs in healthy subjects.	subjects not competitive skiers
Larsson and Larsson 1994	[Better results in sports after use of anti-asthmatic drugs?].	editorial, commentary, interview
Lazovic 2015	Respiratory adaptations in different types of sport	subjects not competitive skiers
Lereim 1993	[Sports in cold climate].	editorial, commentary, interview
Lund 2009	Prevalence of asthma-like symptoms, asthma and its treatment in elite athletes	subjects not competitive skiers
Maiolo 2004	Prevalence of asthma and atopy in Italian Olympic athletes	subjects not competitive skiers
Martinović 2014	Oxidative stress status in female athletes with an IgE-dependent allergic response	subjects not competitive skiers
Michalak 2002	Prevalence of asthma in athletes, influence of sport and environmental exposure	**included in the review**
Millward 2009	The diagnosis of asthma and exercise-induced bronchospasm in division i athletes	subjects not competitive skiers
Mohammadizadeh 2013	The effect of high intensity interval exercise in high/low temperatures on exercise-induced bronchoconstriction (EIB) in trained adolescent males	subjects not competitive skiers
Molphy et al. 2017	The effect of 400 µg inhaled salbutamol on 3 km time trial performance in a low humidity environment	subjects not competitive skiers
Mueller 2001	Immunological effects of competitive versus recreational sports in cross-country skiing	not asthma
Muller 1997	Exercise prescription in cross country skiing for patients with asthma bronchiale	subjects not competitive skiers
Niemelä 2015	Clinical and Laboratory Responses of Cross-Country Skiing for a 24-H World Record: Case Report.	not asthma
Nikitina 2013	Exercise-induced bronchoconstriction (EIB) in different periods of training in winter sports athletes, relationship with respiratory NO production	conference abstract
Nikitina 2014	Efficacy of antileukotriene therapy in exercise-induced bronchospasm in skiers and biathlonists	**included in the review (eventually excluded due to high risk of bias)**
Nikitina ym. 2013	The interaction between respiratory function and exhaled nitric oxide in exercise-induced bronchoconstriction in sportsmen	**included in the review (eventually excluded due to high risk of bias)**
Norqvist 2015	Self-reported physician-diagnosed asthma among Swedish adolescent, adult and former elite endurance athletes.	**included in the review**
Nystad ym. 2000	Asthma and wheezing among Norwegian elite athletes	skiers cannot be separated from other subjects
Ogston and Butcher 2002	A sport-specific protocol for diagnosing exercise-induced asthma in cross-country skiers.	**included in the review**
Oldridge 1981	Cross-country skiing: Precautions for cardiac patients	not asthma
Passàli 2004	Alterations in rhinosinusal homeostasis in a sportive population: Our experience with 106 athletes	not asthma
Petruson 1993	[Facilitate nose breathing during skiing].	editorial, commentary, interview
Pirilä ym. 2000	Nasal function of skiers in cold weather.	not asthma
Pluim ym. 2011	beta(2)-Agonists and Physical Performance A Systematic Review and Meta-Analysis of Randomized Controlled Trials	review
Pohjantähti ym. 2005	Exercise-induced bronchospasm among healthy elite cross country skiers and non-athletic students	**included in the review**
Pollard and Pollard 1994	Asthma in cross country skiers.	editorial, commentary, interview

**Author and year**	**Title**	**Decision**
Price 2016	The role of impulse oscillometry in detecting airway dysfunction in athletes	subjects not competitive skiers
Rundell 2000	Exercise-induced asthma screening of elite athletes: field versus laboratory exercise challenge.	skiers cannot be separated from other subjects
Rundell 2001	Self-reported symptoms and exercise-induced asthma in the elite athlete.	**included in the review**
Rundell 2003	Bronchoconstriction during cross-country skiing: is there really a refractory period?	skiers cannot be separated from other subjects
Rundell 2004	Field exercise vs laboratory eucapnic voluntary hyperventilation to identify airway hyperresponsiveness in elite cold weather athletes.	skiers cannot be separated from other subjects
Rundell 2006	COMMENTARY.	editorial, commentary, interview
Rundell et al. 2018	Exercise-Induced Bronchoconstriction and the Air We Breathe.	review
Sandsund 1998	Effect of cold exposure (-15degreeC) and Salbutamol treatment on physical performance in elite nonasthmatic cross-country skiers	**included in the review**
Sandsund 2000	Treatment with inhaled beta(2)-agonists or oral leukotriene antagonist do not enhance physical performance in nonasthmatic highly trained athletes exposed to-15 degrees C	subjects not competitive skiers
Sandsund 2001	Self-reported asthma and exercise-induced respiratory symptoms related to environmental conditions in marathon runners and cross-country skiers	subjects not competitive skiers
Selge ym. 2016	Asthma prevalence in German Olympic athletes: A comparison of winter and summer sport disciplines	skiers cannot be separated from other subjects
Shephard 2004	Does cold air damage the lungs of winter athletes?	review
SILVERS 1988	The skier's nose: A randomized, double-blind placebo controlled study of ipratropium bromide (AtroventR MDI) administered intranasally for the prevention of cold-induced rhinorrhea	not asthma
Smith 1994	Untitled.	editorial, commentary, interview
Smith 2002	Prevalence of obstructive airflow limitation in Irish collegiate athletes	subjects not competitive skiers
Soligard 2015	Sports injuries and illnesses in the Sochi 2014 Olympic Winter Games	not asthma
Spiering ym. 2004	An evaluation of standardizing target ventilation for eucapnic voluntary hyperventilation using FEV1	subjects not competitive skiers
Stang 2013	Increased Parasympathetic Tone and Bronchial Hyperresponsiveness In Elite Winter Sport Athletes	conference abstract
Stang 2015	Increased bronchial parasympathetic tone in elite cross-country and biathlon skiers: a randomised crossover study.	withdrawn
Stang et al. 2018	The Role of Airway Inflammation and Bronchial Hyperresponsiveness in Athlete's Asthma.	**included in the review**
Stang ym. 2016	Parasympathetic Activity and Bronchial Hyperresponsiveness in Athletes.	not asthma
Steffen 2017	Sports injuries and illnesses in the Lillehammer 2016 Youth Olympic Winter Games	not asthma
Stenfors 2010	Self-reported symptoms and bronchial hyperresponsiveness in elite cross-country skiers.	**included in the review**
Stenfors and Sue-Chu 2008	A comparison of inhaled mannitol, metacholine provocation and eucapnic voluntary hyperventilation as diagnostic tests for exercised induced bronchoconstriction in cross country skiers	conference abstract
Stensrud 2007	Bronchial hyperresponsiveness in skiers: field test versus methacholine provocation?	**included in the review**
Stensrud 2007	Exercise capacity and exercise-induced bronchoconstriction (EIB) in a cold environment	subjects not competitive skiers
Sue-Chu 1996	Prevalence of asthma in young cross-country skiers in central Scandinavia: Differences between Norway and Sweden	**included in the review**
Sue-Chu 1999	Bronchoscopy and bronchoalveolar lavage findings in cross-country skiers with and without "ski asthma".	**included in the review**
Sue-Chu 2000	Placebo-controlled study of inhaled budesonide on indices of airway inflammation in bronchoalveolar lavage fluid and bronchial biopsies in cross-country skiers.	**included in the review**
Sue-Chu 2012	Winter sports athletes: long-term effects of cold air exposure.	review
Sue-Chu ym. 1998	Lymphoid aggregates in endobronchial biopsies from young elite cross-country skiers.	**included in the review**
Sue-Chu ym. 1999	Non-invasive evaluation of lower airway inflammation in hyper-responsive elite cross-country skiers and asthmatics.	**included in the review**

Author and year	Title	Decision
Sue-Chu ym. 1999	Salmeterol and physical performance at -15 degrees C in highly trained nonasthmatic cross-country skiers.	**included in the review**
Sue-Chu ym. 2000	Montelukast does not affect exercise performance at subfreezing temperature in highly trained non-asthmatic endurance athletes.	skiers cannot be separated from other subjects
Sue-Chu ym. 2010	Airway hyperresponsiveness to methacholine, adenosine 5-monophosphate, mannitol, eucapnic voluntary hyperpnoea and field exercise challenge in elite cross-country skiers.	**included in the review**
Svendsen 2016	Training-related and competition-related risk factors for respiratory tract and gastrointestinal infections in elite cross-country skiers	not asthma
Thomas 2010	Self-reported asthma and allergies in top athletes compared to the general population - results of the German part of the GA2LEN-Olympic study 2008.	subjects not competitive skiers
Tjorhom 2007	Effects of formoterol on endurance performance in athletes at an ambient temperature of -20 degrees C	subjects not competitive skiers
Tjørhom 2007	Effects of formoterol on endurance performance in athletes at an ambient temperature of -20°C	not asthma
Tkachenko ym. 1986	[Typological characteristics of the physiological reactions to physical loading in highly trained biathlon athletes].	not asthma
Tornling 1996	Work at low temperature induces inflammation in the lower airways	conference abstract
Tsukioka et al. 2017	Phenotypic analysis of asthma in Japanese athletes	skiers cannot be separated from other subjects
Tucker 1957	The hazards of winter sports.	not asthma
Turcotte 2003	Are questionnaires on respiratory symptoms reliable predictors of airway hyperresponsiveness in athletes and sedentary subjects?	skiers cannot be separated from other subjects
Turmel 2012	Cardiorespiratory Screening in Elite Endurance Sports Athletes: The Quebec Study	**included in the review**
Turmel ym. 2012	Seasonal variations of cough reflex sensitivity in elite athletes training in cold air environment	not asthma
Vakali et al. 2017	Exercice-induced bronchoconstriction among athletes: Assessment of bronchial provocation tests	subjects not competitive skiers
Verges ym. 2004	A 10-year follow-up study of pulmonary function in symptomatic elite cross-country skiers– athletes and bronchial dysfunctions.	**included in the review**
Watts 1993	Physiological responses to specific maximal exercise tests for cross-country skiing.	not asthma
Weiler ym. 2000	Asthma in United States olympic athletes who participated in the 1998 olympic winter games.	skiers cannot be separated from other subjects
Weiler ym. 2016	Exercise-induced bronchoconstriction update-2016	review
Wiggen 2013	Effect of cold conditions on double poling sprint performance of well-trained male cross-country skiers.	not asthma
Wilber ym. 2000	Incidence of exercise-induced bronchospasm in Olympic winter sport athletes.	**included in the review**
Zebrowska 2015	Endurance training and the risk of bronchial asthma in female cross-country skiers.	**included in the review**
Hyrkäs-Palmu 2018	Cold weather increases respiratory symptoms and functional disability especially among patients with asthma and allergic rhinitis	subjects not competitive skiers
Kennedy 2018	Respiratory function and symptoms post cold air exercise in female high and low ventilation sport athletes	subjects not competitive skiers
Kurowski 2018	Serum but not exhaled breath condensate periostin level is increased in competitive athletes	subjects not competitive skiers
Näsman 2018	Asthma and Asthma Medication Are Common among Recreational Athletes Participating in Endurance Sport Competitions	subjects not competitive skiers
Persson 2018	Asthma Control and Asthma Medication Use among Swedish Elite Endurance Athletes.	skiers cannot be separated from other subjects
Skrgat 2018	Systemic and airway oxidative stress in competitive swimmers	subjects not competitive skiers
Tschernig 2018	Isolated aggregates of lymphoid cells in the inner bronchial wall in asthma patients	subjects not competitive skiers
Chernyak et al. 2019	Lung function in athletes involved in skiing and speed skating	not asthma
Kennedy et al. 2019	Cold air exercise screening for exercise induced bronchoconstriction in cold weather athletes.	skiers cannot be separated from other subjects
O'Neill et al. 2019	Asthma prevalence and control levels among Special Olympics athletes, and asthma-related knowledge of their coaches	skiers cannot be separated from other subjects
